# Drug-Coated Balloons for the Treatment of Symptomatic Intracranial High-Grade Stenosis: A Review of the Current Rationale

**DOI:** 10.3389/fneur.2021.692208

**Published:** 2021-07-27

**Authors:** Philipp Gruber, Samarth Singh, Lukas Andereggen, Jatta Berberat, Luca Remonda

**Affiliations:** ^1^Department of Neuroradiology, Kantonsspital Aarau, Aarau, Switzerland; ^2^Department of Neurology, Norvic International Hospital, Kathmandu, Nepal; ^3^Department of Neurosurgery, Kantonsspital Aarau, Aarau, Switzerland; ^4^Faculty of Medicine, University of Bern, Bern, Switzerland

**Keywords:** stroke, intracranial stenosis, intracranial atherosclerotic disease, endovascular procedures, angioplasty, drug-coated balloon

## Abstract

Symptomatic intracranial atherosclerotic disease (sICAD) remains a challenging disorder in the neurovascular field. Despite best medical treatment, the recurrence rate for stroke remains high in patients with intracranial high-grade stenosis (>70–99%). Furthermore, two large randomized trials (SAMMPRIS and VISSIT) failed to prove the efficacy of percutaneous transluminal angioplasty and stenting in patients with sICAD. Drug-coated balloon percutaneous transluminal angioplasty (DCB-PTA) represents an alternative treatment modality with therapeutic benefits for interventional cardiology. However, there are very few articles in the existing literature that relate to the use of DCB-PTA in sICAD patients. Here, we aimed to review the rationale underlying the use of DCB-PTA in sICAD patients and summarize recent developments in the neurovascular field.

## Introduction

Intracranial atherosclerotic disease (ICAD) is a common global disease. Symptomatic ICAD (sICAD) is known to cause 10% of all transient ischemic attacks and strokes worldwide (1, 2). The treatment of sICAD remains challenging given that the recurrence rate of strokes can reach up to 38.2% of patients despite best medical treatment (BMT) (3). The risk of stroke recurrence is particularly high in patients with hemodynamically relevant stenosis or unstable atherosclerotic plaques (3). Current guidelines recommend BMT, a combination of anti-platelet therapy and vascular risk factor control, as first-line therapy (4, 5), with endovascular therapies considered as rescue therapies (4, 5).

The “Warfarin-Aspirin Symptomatic Intracranial Disease” (WASID) trial demonstrated that oral anticoagulation (warfarin) was not superior to aspirin as a single therapy for ICAD patients (6). Subsequently, the findings of the “Clopidogrel plus aspirin vs. aspirin alone for reducing embolization in patients with acute symptomatic cerebral or carotid artery stenosis” (CLAIR) trial and the “Clopidogrel and Aspirin for Reduction of Emboli in Symptomatic Carotid Stenosis” (CARESS) trial provided the rationale behind the use of short-term dual antiplatelet therapy for patients with ICAD (7, 8).

The successful use of percutaneous transluminal angioplasty (PTA) to treat symptomatic, high-grade, basilar stenosis was first reported in 1980 (9). In the early 2000s, the publication of numerous case series clearly demonstrated the feasibility of applying PTA as a single therapy, or in combination with stenting (PTAS), for patients with ICAD (10, 11). With regards to stroke and death rates, the outcomes of PTA tend to vary widely (4–40%) within 30 days of treatment; in addition, 24–50% of patients undergoing PTA developed restenosis (12). Furthermore, dissection and immediate re-coiling can occur during the PTA technique. The results published by the WASID trial were not encouraging (6); consequently, the use of PTAS was strongly encouraged. The single-arm “Stenting of Symptomatic Atherosclerotic Lesions in the Vertebral or Intracranial Arteries” (SSYLVIA) trial subsequently provided convincing data to support the use of a stent system to treat symptomatic intra- and extracranial stenosis (13). In addition, two different cohort studies reported promising results for the use of a novel self-expanding Wingspan stent system (14, 15). Therefore, the “Stenting vs. Aggressive Medical Management for Intracranial Arterial Stenosis” (SAMMPRIS) trial was initiated to specifically compare aggressive medical treatment (AMM) with PTAS using the Gateway Balloon PTA system combined with the Wingspan stent (Stryker, Kalamazoo, USA) (16). The SAMMPRIS trial failed to demonstrate any superiority of the PTAS treatment in comparison with AMM due to the discovery of a significantly higher risk of early ischemia within 30 days in the PTAS group (14.7%) compared with an AMM only group (5.8%) (16). Furthermore, the PTAS group was dominated by peri-interventional complications. Shortly thereafter, the SAMMPRIS data were confirmed by the “Vitesse Intracranial Stent Study for Ischemic Stroke Therapy” (VISSIT) trial, which compared BMT with a balloon-expandable stent system and found that the two techniques were similar with regards to outcome (17). The long-term results provided by the SAMMPRIS trial highlighted the early benefit of AMM compared with PTAS in patients with high-grade ICAD; this effect persisted over an extended median follow-up period of 32.4 months (18). As a consequence, there was a significant decline in the use of endovascular treatment to treat patients with sICAD. Nevertheless, there is an ongoing debate relating to the use of endovascular treatment for patients with sICAD (19, 20).

Short-term results derived from a Chinese multicenter registry study (*n* = 300) revealed a stroke, bleeding, and death rate of only 4.5% of patients (21). The on-label, multicenter “Wingspan Stent System Post Market Surveillance” (WEAVE) trial demonstrated that the peri-interventional complication rate of PTAS in ICAD patients decreased to only 2.6% in centers with experienced interventionalists and rigorous patient selection protocol (22). Furthermore, the longer-term (1-year) “Wingspan One-year Vascular Events and Neurologic Outcomes” (WOVEN) trial reported sustained benefit for the PTAS group (23). The “China Angioplasty and Stenting for Symptomatic Intracranial Severe Stenosis” (CASSISS) trial, involving the Wingspan stent system, presented their preliminary results at the 14th World Federation of Interventional Radiology and Therapy: stroke or death only occurred in 2% of patients (24, 25). In addition, other self-expanding stent systems such as the Enterprise (Codman, Raynham, USA) or Neuroform stents (Stryker Neurovascular, Fremont, USA) showed in several series promising results in the treatment of symptomatic ICAD patients (26, 27). The introduction of the first-balloon-then-stent technique with the novel self-expanding Credo stent (Acandis, Pforzheim, Germany) together with the NeuroSpeed balloon-catheter system (Acandis, Pforzheim, Germany) may further reduce the perinterventional complication rate (28). In a cohort of 76 ICAD patients treated with another new-generation, self-expanding stent system [Acclino stent/NeuroSpeed balloon catheter system (Acandis, Pforzheim, Germany)], feasibility and safety were promising with a periprocedural stroke rate of 6.5% (29). Thus, novel stent technologies as well as new procedural techniques may further reduce the overall morbidity and mortality in ICAD stenting in the future.

## Restenosis: a Common Long-term Sequela in Endovascular Therapy

In addition to peri-procedural complications, restenosis is frequently observed as a long-term sequela, both in PTA and PTAS. In PTA, restenosis rate within 6 months was reported in 5–30% of cases (30). A similar proportion of patients (25%) experienced in-stent-restenosis following PTAS (31). The precise mechanisms underlying these findings have yet to be elucidated; however, restenosis is mainly caused by neointimal hyperplasia (NIH), a condition that is induced by mechanical stress and endothelial lesions during PTA and PTAS (32). There are several risk factors for NIH, including age, diabetes mellitus, lesion location, and a history of active smoking (33). NIH is a frequent long-term problem associated with peripheral PTA and interventional cardiology. Consequently, drug-eluted stents (DES) and drug-coated balloons (DCB) were introduced to overcome this issue. In addition, a range of drugs are now available to prevent NIH. Mitotic inhibitors (e.g., paclitaxel) or immuno-modulators (e.g., sirolimus) are commonly used for DES and DCB-PTA coating. DES and DCB-PTA are frequently used in interventional cardiology and have been found to be both safe and effective (34).

Publications relating to the use of DCB-PTA and DES in patients with ICAD are scarce. With the introduction of stent-assisted intracranial stenosis treatment, the use of DES has been shown to be both safe and efficient (35, 36). Similarly, studies have shown that the use of coronary DES for sICAD is feasible and safe. However, a high rate of technical failure has been reported due to DES stiffness. An improved stent deployment rate was achieved using a more flexible DES (37). A study of sICAD patients treated by DCB and the deployment of a bare-metal stent revealed encouraging results with a low rate of restenosis rate (3%) (38).

## Drug-Coated Balloons in the Treatment of Symptomatic ICAD

DCB-PTA may represent a promising alternative to PTA or PTAS for the treatment of patients with ICAD (39). DCB-PTA has the potential to minimize peri-interventional and long-term complication rates in the endovascular treatment. In the tortuous neurovascular anatomy, DCB-PTA is more flexible due to a softer distal PTA tip compared with DCS, thus enabling the operator to reach more distant lesions. Endovascular DCB-PTA procedures do not leave residual foreign bodies, thus exerting a positive impact on the possibility of subsequent adverse material-tissue reactions and local flow dynamics (40). In contrast to DES, DCB-PTA offers a uniform anti-proliferative drug coverage of the diseased vessel lumen. Furthermore, a shorter duration of recommended dual anti-platelet therapy (DAPT) might be reasonable for DCB-PTA given the lower risk of delayed endothelialization and subsequent thrombosis when compared with DES (41).

Since 2018, various retrospective and comparative cohort and single studies of DCB-PTA for the treatment of sICAD patients have been published (42–47) (Table 1). These studies featured a range of different DCB-PTA systems, including the Neuro Elutax SV (Aachen Resonance, Aachen, Germany), the Sequent Please (B Braun, Melsungen, Germany), and the Sequent Please NEO (B Braun). All of these DCBs were coated with paclitaxel, a highly lipophilic mitotic inhibitor. These studies also described a range of different DCB-PTA procedures. Three (*n* = 6) of these studies reported the use of only submaximal angioplasty using a DCB-PTA (42, 44, 47). The other three studies pre-dilated the target lesion using a non-coated PTA balloon (Gateway balloon) immediately followed by DCB-PTA (Sequent Please) (43, 45, 46). When comparing the final post-procedural degree of stenosis, the DCB-PTA only group revealed a higher degree of residual stenosis (37.5–50%) compared with the combined PTA/DCB-PTA group (10–32%). However, there is a lack of systematic data that could demonstrate which method is superior with regards to short- and longer-term outcome. One advantage of the DCB-PTA only approach over the combined approach is that the numbers of intracranial maneuvers can be reduced, whereas the combined approach affords at least one additional step (the exchange of PTA to a DCB-PTA).

**Table 1:** An overview of existing drug-coated balloon percutaneous transluminal angioplasty (DCB-PTA) studies in patients with symptomatic high-grade intracranial stenosis.

**Study group**	No. of patients^*^	**DCB type**	**Follow-up period in months**	**Degree of pretreatment stenosis (%)**	DCB-PTA only^*^	**Degree of post-treatment stenosis (%)**	**Complications** No. (%)^**^	**Restenosis** No. (%)^**^	**Symptomatic** **restenosis** No. (%)^**^
Gruber et al. (42)	8^†^	Neuro Elutax SV	9.5	80	Yes	37.5%	0	1 (13%)	0
Han et al. (43)	30	Sequent Please	9.8	82%	No	20%	2 (3.2)	1 (3.2)	0
Gruber et al. (44)	10	Sequent Please NEO	3	78%	Yes	50%	0	0	0
Zhang et al. (45)	42^††^	Sequent Please	6	90%	No	10%	4	2 (4.8%)	1 (2.4%)
Wang et al. (46)	35	Sequent Please	20.9	76.6%	No	32.4%	4 (11.4%)	3 (8.3%)	0
Remonda et al. (47)	33	Neuro Elutax SV/Sequent Please NEO	9	80%	Yes	50%	3 (9%)	5 (15%)	4 (12%)

* *without pre-dilation using another PTA balloon system (Gateway balloon)*;

** *No, number*;

† *comparative study with a total number of 19 patients*;

†† *comparative study with a total number of 115 patients*.

Further analysis showed that the mean follow-up period was variable and ranged from 3 to 21 months, while follow-up was heterogeneously defined. Two factors that were common to all of these DCB-PTA studies were an overall low complication rate and promising results with regards to symptomatic and asymptomatic restenosis rates.

In their series of sICAD patients treated with either Sequent Please NEO alone (*n* = 10) or Neuro Elutax SV and Sequent Please NEO (*n* = 33), Gruber et al. reported promising short- (a median of 3 months) and mid-term results (a median of 9 months) with only few symptomatic cases of restenosis (12%) along with low rates of intracranial complications (6%) (44, 47). Another study of sICAD patients (*n* = 30), treated with routine PTA followed by additional DCB-PTA, reported similar results with regards to restenosis and complication rates (43). A recent Chinese study (*n* = 35), using the Sequent Please DCB-PTA procedure, reported low complications and a low recurrence rate (stenosis >50%) (45).

Two retrospective comparative studies of DCB-PTA and PTAS provided further support to the findings of the mono-cohort studies (45, 46). A small, single-center, retrospective study (*n* = 19) comparing DCB-PTA and PTAS using the Wingspan Stent system demonstrated a lower asymptomatic and symptomatic restenosis rate compared with the PTAS group (41) although there was no difference between the two techniques with regards to complication rates. In a recently published Chinese study (*n* = 115), the restenosis rate was significantly lower in the DCB-PTA group than in a PTAS group (45). However, there were no differences between the two groups with regards to safety and the recurrence of stroke (45). Overall, the technical success rate was reported to be high, although in one study, DCB-PTA could not be advanced over the lesion due to difficult local anatomy (42); two other studies reported few bail-out maneuvers with PTAS (43, 46). The most common periprocedural complication was ischemic events.

In summary, all of these studies demonstrated promising results for symptomatic intracranial high-grade stenosis. However, there is a lack of prospective data with regards to long-term results.

## Future Direction and Concluding Remarks

It is clear that DCB-PTA offers an alternative treatment option for patients with sICAD compared with BMT and the other endovascular procedures that are used at present. Recent studies have demonstrated encouraging results regarding the use of DCB-PTA, with low complication and restenosis rates. However, future studies should address several key questions. For example, is submaximal DCB-PTA alone superior to the combination of PTA followed by DCB-PTA? Is paclitaxel the appropriate choice for drug coating or are other coating strategies more beneficial for neurovascular applications? Furthermore, we need to be able to identify the ICAD patients for whom DCB-PTA would be the most suitable treatment option. Randomized trials may shed light on whether DCB-PTA is superior to BMT or PTAS in patients with sICAD.

In conclusion, DCB-PTA is a feasible procedure for the treatment of patients with sICAD and represents a promising treatment modality for the future treatment of ICAD. However, further prospective data are now needed to validate the precise role of DCB-PTA in sICAD.

## Author Contributions

PG helped to conceive and design this research, acquired, analyzed, interpreted the data, and wrote the article. SS helped to conceive and design the research and revised the article for important intellectual content. JB and LA revised the article for important intellectual content. LR helped to conceive and design this research, revised the article for important intellectual content, and approved the final version of the article for publication. All authors contributed to the article and approved the submitted version.

## Conflict of Interest

The authors declare that the research was conducted in the absence of any commercial or financial relationships that could be construed as a potential conflict of interest.

## Publisher's Note

All claims expressed in this article are solely those of the authors and do not necessarily represent those of their affiliated organizations, or those of the publisher, the editors and the reviewers. Any product that may be evaluated in this article, or claim that may be made by its manufacturer, is not guaranteed or endorsed by the publisher.
